# The Role of Social Support for Depressive Symptoms in Dementia: A Four-Year Longitudinal Study

**DOI:** 10.1093/geroni/igaf047

**Published:** 2025-07-01

**Authors:** Iris Blotenberg, Lina Jeran, Francisca S Rodriguez, Bernhard Michalowsky, Moritz Platen, Stefan Teipel, Wolfgang Hoffmann, Jochen René Thyrian

**Affiliations:** German Center for Neurodegenerative Diseases (DZNE), Greifswald, Germany; German Center for Neurodegenerative Diseases (DZNE), Greifswald, Germany; German Center for Neurodegenerative Diseases (DZNE), Greifswald, Germany; German Center for Neurodegenerative Diseases (DZNE), Greifswald, Germany; German Center for Neurodegenerative Diseases (DZNE), Greifswald, Germany; German Center for Neurodegenerative Diseases (DZNE), Rostock, Germany; Department of Psychosomatic Medicine, University Medicine Rostock, Rostock, Germany; German Center for Neurodegenerative Diseases (DZNE), Greifswald, Germany; Institute for Community Medicine, University Medicine Greifswald, Greifswald, Germany; German Center for Neurodegenerative Diseases (DZNE), Greifswald, Germany; Institute for Community Medicine, University Medicine Greifswald, Greifswald, Germany

**Keywords:** Alzheimer’s disease, Mental health, Neurodegenerative diseases, Neuropsychiatric symptoms, Social health

## Abstract

**Background and Objectives:**

Depressive symptoms are common in people with dementia, significantly reducing well-being and potentially exacerbating dementia symptoms. The objective of the present study was to investigate the role of support from the social environment for depressive symptoms in people with dementia over a 4-year period.

**Research Design and Methods:**

We used data from a cohort of 334 community-dwelling people with dementia (*M*_age_ = 80.2, 59.3% female) who were interviewed annually in their homes by specially qualified nurses. We used multilevel growth curve models with random intercepts and slopes to model depressive symptoms over time. We modeled both the role of between-person differences and the role of within-person changes in social support for depressive symptoms.

**Results:**

At the beginning of the study, 13.8% of people with dementia reported mild to severe depressive symptoms. People with more social support showed fewer depressive symptoms overall over the 4-year period (% change per point on a scale from 22 to 110: −1.2, 95% CI: −1.8, −0.4). In addition, a decline in a person’s social support was associated with more depressive symptoms (% change: −0.9, 95% CI: −1.7, −0.2). These effects were stable even after controlling for sociodemographic (age, sex, education) and clinical factors (cognitive and functional status, comorbidities).

**Discussion and Implications:**

The social environment plays an important role in depressive symptoms in people with dementia—beyond clinical factors like cognitive and functional abilities. Improving support from the social environment could be a lever for alleviating depressive symptoms. In the care of people with dementia, not only medical needs but also psychosocial needs should come to the forefront.

**Translational Significance:**

To date, little research has been conducted on the factors that influence depressive symptoms in people with dementia. In this longitudinal study, we show for the first time that the extent of support from the social environment is associated with depressive symptoms, even after controlling for clinical variables. Enhancing social support is likely to be an effective approach for reducing depressive symptoms in people with dementia.

## Background and Objectives

Dementia is one of the major public health challenges of our time. Globally, the number of people living with dementia was estimated at 50 million in 2017, and in aging societies worldwide, the number is rising (Blotenberg et al., 2023; World Health Organization, 2021). Dementia is one of the leading causes of care dependency and disability in older age. The condition presents significant challenges for individuals living with dementia, their families, and healthcare systems. These challenges are further aggravated by a shortage of caregivers and the increasing strain on health care systems. In 2019, the global direct and indirect costs of dementia were estimated at $1.3 trillion (World Health Organization, 2021).

In addition to the hallmarks of the syndrome—cognitive decline and decline in everyday function—people with dementia also frequently experience depressive symptoms. Depending on the type and severity of dementia, prevalence estimates vary. A meta-analysis reported an average prevalence of 15.9% for major depressive disorder (Asmer et al., 2018). Depression can exacerbate dementia symptoms and significantly reduce well-being (Blotenberg et al., 2025; Lyketsos & Olin, 2002). In addition, depressive symptoms in people with dementia increase caregiver burden and the likelihood of admission to a nursing home (Lyketsos & Olin, 2002). To date, only a limited number of studies have investigated the factors associated with depressive symptoms in people with dementia, and the findings are heterogeneous (Steck et al., 2018). Furthermore, the focus has primarily been on the biological causes of depression in Alzheimer’s disease and related dementias, which are linked to neurodegenerative and pathological changes in the brain (Bennett & Thomas, 2014; Olin et al., 2002).

Studies show that the social environment plays a significant role in depressive symptoms among older adults. In a systematic review, Gariépy et al. (2016) highlighted a general link between social support and depression in this population. Similarly, Schwarzbach et al. (2014) examined the influence of different aspects of social relationships on depressive symptoms in older adults. Their findings revealed that qualitative aspects of relationships, such as social support, the quality of interactions, and the presence of confidants, showed more consistent protective effects against depression compared to quantitative aspects. Yet, it is unclear whether similar associations can be found in older adults with dementia, as depression that co-occurs with dementia differs from depression in people without neurological cognitive impairment (Olin et al., 2002).

The role of the social environment in the development and progression of depressive symptoms in people with dementia has received little attention so far. However, it is well-established that social isolation is an important risk factor for dementia (Samtani et al., 2022). Moreover, the COVID-19 pandemic highlighted the importance of the social environment for the overall health and well-being of people with dementia, as restrictions on social contacts led to worsening of both cognitive and psychological symptoms (Giebel et al., 2023). In this context, the functional aspect of the social environment—namely, social support—is of particular interest. Social support may be defined as the “perception or experience that one is cared for, esteemed, and part of a mutually supportive network” (Taylor, 2011). It is considered one of the key mechanisms through which the social environment exerts its beneficial effects on health and well-being (Berkman et al., 2000 ; Vila, 2021). A recent study demonstrated that people with dementia who report greater perceived social support have a one-year longer life expectancy compared to those with lower levels of support (Blotenberg et al., 2024).

Given the cognitive and functional impairments associated with dementia, active social engagement and network-building may be particularly challenging. This underscores the importance of external social support as a modifiable factor that can be actively provided, rather than relying on self-initiated social engagement. Therefore, social support could represent a particularly promising target for interventions aimed at mitigating depressive symptoms and improving well-being in people with dementia.

### The Present Study

In the present study, we used data from community-dwelling people screened positive for dementia who were visited and comprehensively interviewed annually by specially qualified nurses. We examined whether more social support was associated with fewer depressive symptoms over a four-year period, after controlling for sociodemographic and clinical variables. We not only examined interindividual differences (i.e., people with more compared to less social support) but also the role of intraindividual increases or decreases of social support and its association with depressive symptoms.

## Research Design and Methods

### Sample and Study Procedure

For the present study, data from the cluster-randomized DelpHi-MV trial (Dementia: Life- and Person-centered Help in Mecklenburg-Western Pomerania, Germany) were used (Thyrian et al., 2017). Originally, the study was conducted to evaluate the effectiveness of Dementia Care Management (DCM) compared to care as usual after one year. DCM is a six-month intervention in which specially qualified nurses support community-dwelling people with dementia in obtaining optimal care and support. After the effectiveness study was completed, the sample was continued to be monitored (Michalowsky et al., 2024). The study received ethical approval from the ethics committee of the Chamber of Physicians of Mecklenburg-Western Pomerania—registry number: BB 20/11.

For the study, 6,838 community-dwelling patients were screened for eligibility at one of 125 participating general practitioners’ (GPs) practices. Of these, 1,166 people met the eligibility criteria, namely, they were screened positive for dementia (DemTect score <9), lived at home, and were at least 70 years old. Of these, 634 people provided informed consent for participation in the study. A few months after study enrolment (median: 3 months), the baseline assessment followed in the participants’ homes. Specially qualified nurses recorded extensive information on cognitive status, daily functioning, the care situation, and psychosocial factors in a structured interview. In the further course of the study, the patients were visited and extensively interviewed annually. This longitudinal study comprises five measurements over a period of four years. It is based on data from 334 people who had answered the questions on depressive symptoms on at least two measurement occasions and who had answered the questions about social support on at least one occasion. The participant flow over four years is shown in Supplementary Figure 1 (Supplementary Material).

### Measures

#### Primary outcome

*Depressive symptoms* were assessed annually using the 15-item Geriatric Depression Scale (GDS; Yesavage et al., 1982) . The questionnaire’s sensitivity and specificity for detecting depressive symptoms in people with dementia have been demonstrated (Park, 2022). The sum score can range from 0 to 15 with higher values indicating more depressive symptoms (>5 points: mild to moderate depression, >10 points: severe depression). For the sensitivity analysis with the categorical dependent variable depression (yes/no), we transformed the GDS score into a binary variable with the categories “no indication of depression” (GDS <5 points) and “indication of depression” (GDS ≥5 points).

#### Predictors

*Social support* was assessed annually using the FSozU K-22 (“Questionnaire for the assessment of social support”; Fydrich et al., 2007). The 22-item questionnaire measures perceived social support from family, friends, and acquaintances. It has been extensively validated and used both in the general population and in clinical studies (Fydrich et al., 2007). Example items are: “There are people who accept me as I am,” “I have friends/family who are good at listening when I need to talk,” “When I am sick, I can ask friends or family members to help me with important tasks (e.g., shopping) without hesitation.” or “There is a community of people (circle of friends, group) to which I feel a sense of belonging.” The items were answered on a five-point Likert scale, where 1 represents “*does not apply*” and 5 represents “*exactly applicable*.” The sum score was calculated from all items, higher scores indicate more social support.

*Cognitive status* was assessed annually using the Mini-Mental State Examination (MMSE; Kessler et al., 1990), which covers a range of cognitive abilities, such as orientation, recall, attention, and calculation. It yields scores from 0 to 30; scores of 27 and above indicate normal cognitive performance, scores between 20 and 26 indicate mild cognitive impairment, scores between 10 and 19 indicate moderate dementia, and scores below 10 indicate severe dementia.

*Functional status* was assessed annually using the Bayer Activities of Daily Living scale (B-ADL; Hindmarch et al., 1998), which covers a range of daily problems from problems with personal care to problems with shopping or meal preparation. It yields scores ranging from 1 to 10 and was recoded so that higher values indicate higher functional status.

#### Covariates

We adjusted for sociodemographic factors such as age, sex, education, and living situation, as well as for comorbidities, because these factors may be associated with both the social environment and depressive symptoms. Additionally, we controlled for the assignment to the intervention or control group, as this study was originally a cluster-randomized trial, and group allocation could have influenced the care of the person with dementia, potentially affecting symptoms, including neuropsychiatric symptoms like depression. We also controlled for the presence of a caregiving network to examine whether it was support from the social environment or rather structural characteristics such as the network itself that played a role in depressive symptoms.

*Sociodemographic information (age, sex, education, living situation)* was recorded at baseline.

*Educational attainment* was recorded and converted into the classification according to the International Standard Classification of Education (ISCED; UNESCO, 2003). Level 1 (“low education”) corresponds to primary education (elementary school at most), level 2 (“moderate education”) corresponds to lower secondary education (e.g., elementary or lower secondary school, polytechnic secondary school), and level 3 (“high education”) corresponds to upper secondary education (e.g., Abitur).

*Group allocation* (DCM vs. care as usual) was determined by the study center upon inclusion of the study.

To assess the *caregiving network*, it was recorded whether nonprofessional individuals (family members, neighbors, and acquaintances) performed caregiving and noncaregiving tasks (e.g., assistance with shopping), and if that was the case, it was coded that a caregiving network existed.

*Patients’ comorbidities* at baseline were extracted from their medical records to calculate the Charlson comorbidity index. The index ranges from 0 to 37, where higher values indicate a greater comorbidity burden (Charlson et al., 1987).

### Statistical Analysis

The analyses were performed in R (R Core Team, 2022). The significance level was set at *p* < .05. The number of missing values in the variables recorded at baseline was low, with most missing values occurring in the variable caregiving network (13 cases, 3.9%). In the time-varying variables, the number of missing values increased over time. For example, for the dependent variable depressive symptoms: at the first follow-up, 4 cases (1.2%); at the second follow-up, 45 cases (13.5%); at the third, 112 cases (33.5%); and at the fourth follow-up, 188 cases (56.3%) were missing. Missing values were handled using multiple imputations with chained equations. Fifty data sets were created in fifty iterations each.

Multilevel growth curve models were calculated to predict depressive symptoms over the four years. We decided on this statistical model instead of a fixed-effects panel regression model because we were interested in both within-person effects and between-person effects. Additionally, we aimed to account for the clustering within the recruiting general practices, as well as variability between individuals. Multilevel growth curve models are particularly well-suited for this purpose due to their flexibility. Clustering of the data regarding treating GP was taken into account with a random intercept. Starting with the null model, linear and quadratic time variables, as well as random intercepts (for individuals and GP) and slopes were included successively, and models were compared in terms of fit (Akaike Information Criterion, Bayesian Information Criterion).

As depressive symptoms, measured with the GDS, were right-skewed, they were log-transformed. Social support, cognitive status, and functional status were included in the models as time-varying variables. For variables collected at multiple time points, two types of associations with the outcome were of interest—between-person differences and within-person variability. Therefore, the variance was decomposed into two parts: Firstly, the association of between-person differences with depressive symptoms was examined (e.g., the question of whether depressive symptoms were less pronounced in a person with a high level of social support than in a person with a low level of social support). In order to model these differences between individuals, the individual mean value across the five measurements was used. Secondly, the changes within a person over time were considered (e.g., how changes in a person’s social support were associated with their depressive symptoms). For this purpose, the value observed for a person at the respective measurement time was subtracted from the individual mean value. The same approach was used for the time-varying variables of cognitive status and functional status.

### Sensitivity Analysis

To assess the robustness of the results, all analyses were also conducted using the original data without imputations. As an additional sensitivity analysis, depression was predicted as a binary variable (depression yes/no).

## Results

### Descriptive Statistics

Table 1 shows the descriptive statistics for the sample. The study participants were, on average, 80.2 years old (*SD* = 5.3). There were more women than men in the sample (59.3%), and 18.4% had a low, 78.6% a medium, and 3.0% a high level of education. At baseline, 13.8% of the sample reported symptoms of mild to severe depression according to the GDS. The mean MMSE score was 23.1 (*SD* = 4.5), and thus in the range of mild dementia.

**Table 1.** Description of Participant Characteristics at Baseline

Variable	*N*	*M* (*SD*)	*n* (%)
Age in years	334	80.2 (5.3)	
Sex	334		
Female			198 (59.3)
Living situation	334		
Living alone			175 (52.4)
Group allocation	334		
Intervention group			240 (71.9)
Caregiving network	321		
Network available			311 (96.9)
Education	332		
Low			61 (18.4)
Moderate			261 (78.6)
High			10 (3.0)
Charlson comorbidity index (scale: 0–37)	334	3.5 (2.3)	
Cognitive status (scale: 0–30)			
At baseline	328	23.1 (4.5)	
At follow-up 1	315	22.0 (5.4)	
At follow-up 2	263	20.8 (6.1)	
At follow-up 3	181	20.2 (6.7)	
At follow-up 4	127	19.6 (7.6)	
Functional status (scale: 1–10)			
At baseline	330	7.7 (2.3)	
At follow-up 1	319	6.7 (2.7)	
At follow-up 2	287	5.9 (3.1)	
At follow-up 3	227	5.5 (3.1)	
At follow-up 4	156	5.3 (3.1)	
Social support (scale: 22–110)			
At baseline	286	89.7 (13.2)	
At follow-up 1	299	87.8 (13.3)	
At follow-up 2	229	86.8 (14.7)	
At follow-up 3	174	87.0 (14.0)	
At follow-up 4	107	84.0 (13.4)	
Depressive symptoms (scale: 0–15)			
At baseline	334	3.2 (2.4)	
No symptoms			288 (86.2)
Mild to moderate			42 (12.6)
Severe			4 (1.2)
At follow-up 1	330	3.2 (2.6)	
No symptoms			277 (83.9)
Mild to moderate			47 (14.2)
Severe			6 (1.8)
At follow-up 2	289	3.3 (2.5)	
No symptoms			242 (83.7)
Mild to moderate			46 (15.9)
Severe			1 (0.3)
At follow-up 3	222	3.4 (2.6)	
No symptoms			186 (83.8)
Mild to moderate			31 (14.0)
Severe			5 (2.3)
At follow-up 4	146	3.7 (2.7)	
No symptoms			116 (79.5)
Mild to moderate			28 (19.2)
Severe			2 (1.4)

*Notes*: *M* = mean; *SD* = standard deviation.

### Social Support as a Predictor of Depressive Symptoms

The model with the best fit incorporated a linear time variable, along with random intercepts (for individuals and GP) and a random slope. Table 2 displays the parameter estimates for the null model (Model 1). There was a nonsignificant trend toward an increase in depressive symptoms in the sample by 3.9% (95% CI: −1.0, 8.9, *p* = .117) per year.

**Table 2.** Prediction of Depressive Symptoms Across Four Years

Variable	Estimate (log)			% change			*p*
	Est.	95% CI (lower)	95% CI (upper)	Est.	95% CI (lower)	95% CI (upper)	
*Model 1. Null model*							
Fixed effects							
Intercept	1.241	1.161	1.321	–	–	–	<.001***
Time	0.038	−0.010	0.085	3.873	−0.956	8.882	.117
Random effects							
Intercept (person)	0.22						
Time	0.02						
Intercept (person) × Time	−0.03						
Intercept (GP)	0.01						
Residual	0.23						
ICC	0.60						
Marginal *R*^2^/conditional *R*^2^	0.007/0.611						
*Model 2. Full model*							
Fixed effects							
Intercept	2.772	1.509	4.034	−	−	−	<.001***
Time	−0.164	−0.835	0.508	−15.126	−56.621	66.171	.630
Age	−0.002	−0.016	0.011	−0.200	−1.566	1.147	.752
Sex (ref: male)	0.014	−0.130	0.157	1.410	−12.158	16.963	.853
Living alone (ref: no)	−0.035	−0.173	0.104	−3.439	−15.901	10.972	.625
Moderate education (ref: low)	−0.056	−0.229	0.116	−5.446	−20.454	12.336	.523
High education (ref: low)	−0.065	−0.463	0.333	−6.293	−37.041	39.476	.749
Group allocation (ref: CAU)	−0.002	−0.147	0.143	−0.200	−13.661	15.413	.981
Caregiving network (ref: none)	−0.223	−0.624	0.177	−19.989	−46.401	19.370	.274
Charlson comorbidity index	0.026	−0.003	0.054	2.634	−0.294	5.598	.079
Cognitive status (between-person differences)	0.028	0.005	0.050	2.840	0.489	5.161	.018*
Cognitive status (within-person variability)	0.007	−0.014	0.028	0.702	−1.411	2.829	.522
Functional status (between-person differences)	−0.111	−0.153	−0.069	−10.506	−14.197	−6.643	<.001***
Functional status (within-person variability)	−0.053	−0.094	−0.013	−5.162	−8.967	−1.249	.011*
Social support (between-person differences)	−0.012	−0.019	−0.004	−1.193	−1.848	−0.436	.002**
Social support (within-person variability)	−0.009	−0.017	−0.002	−0.896	−1.683	−0.181	.015*
Time × age	0.003	−0.005	0.010	0.300	−0.476	0.988	.494
Time × sex	−0.024	−0.095	0.048	−2.371	−9.066	4.920	.517
Time × living alone	−0.020	−0.087	0.046	−1.980	−8.306	4.713	.547
Time × moderate education	0.009	−0.073	0.092	0.904	−7.036	9.591	.824
Time × high education	−0.013	−0.193	0.167	−1.292	−17.526	18.185	.889
Time × group allocation (ref: CAU)	−0.010	−0.075	0.054	−0.995	−7.205	5.526	.748
Time × caregiving network	0.049	−0.128	0.225	5.022	−11.984	25.259	.587
Time × Charlson comorbidity index	0.002	−0.014	0.017	0.200	−1.390	1.730	.841
Time × cognitive status (between-person differences)	−0.002	−0.012	0.009	−0.200	−1.222	0.926	.777
Time × cognitive status (within-person variability)	−0.002	−0.010	0.006	−0.200	−1.042	0.628	.618
Time × functional status (between-person differences)	0.021	0.000	0.041	2.122	0.048	4.157	.045*
Time × functional status (within-person variability)	0.008	−0.014	0.031	0.803	−1.438	3.151	.472
Time × social support (between-person differences)	−0.002	−0.005	0.001	−0.200	−0.535	0.124	.219
Time × social support (within-person variability)	0.000	−0.004	0.004	0.000	−0.434	0.447	.980
Random effects							
Intercept (person)	0.16						
Time	0.02						
Intercept × Time	−0.02						
Intercept (GP)	0.00						
Residual	0.22						
ICC	0.40						
Marginal *R*^2^/Conditional *R*^2^	0.179/0.525						

*Notes*: CI = confidence interval; ICC = intraclass correlation coefficient; GP = general practitioner. *p <.05, **p <.01, ***p <.001.

Table 2 (Model 2) shows that more social support was significantly linked to fewer depressive symptoms, both in terms of interindividual differences and intraindividual changes over time. With each additional point in social support (range from 22 to 110), depressive symptoms were 1.2% lower (95% CI: −1.8, −0.4). Individual changes in social support also played a role: If a person’s social support increased by one point, depressive symptoms decreased by 0.9% (95% CI: −1.7, −0.2).

### Further Predictors of Depressive Symptoms

People who were cognitively fitter overall showed more depressive symptoms, by 2.8% for each additional point on the MMSE (95% CI: 0.5, 5.2). Fluctuations in cognitive status were not associated with changes in depressive symptoms. Everyday functioning was also associated with depressive symptoms, with both interindividual differences and individual variations playing an important role: people who had better overall everyday functioning had fewer depressive symptoms by 10.5% for each additional point on the ten-point scale of everyday functioning (95% CI: 14.2, 6.6). In addition, people whose everyday function deteriorated during the study period showed, overall, more depressive symptoms; with each point less on the daily functioning scale, depressive symptoms increased by 5.2% (95% CI: 9.0, 1.2). There was an interaction between the time variable and between-person differences in functional status; the difference in depressive symptoms between people with higher and lower functional status slightly decreased over time (2.1%, 95% CI: 0.0, 4.2). Except for that, no significant interactions were found for cognitive and functional status with the time variable, indicating that the effects were stable over time.

### Sensitivity Analysis

Sensitivity analyses based on the original, unimputed data yielded very similar results as the analyses based on the imputed data (see Supplementary Table 1). The second sensitivity analysis, with depression as a categorical variable, is presented in Supplementary Table 2. Here, too, the effects observed in the main analysis remain robust.

## Discussion

The aim of the present study was to explore the role of social support for depressive symptoms in dementia over a four-year study period. Our findings suggest a significant role of the social environment: We demonstrated that lower levels of social support (between-person differences) were associated with more depressive symptoms and that a decline in social support (within-person changes) was associated with an increase in depressive symptoms in people with dementia beyond clinical factors such as cognitive and functional status or comorbidities.

Social support is widely recognized as a protective factor for physical and mental health and theoretical models provide insights into how it may exert its effects (Cohen et al., 2000). According to the “direct-effect model,” social support has a direct protective effect by fulfilling the human need for closeness. Another pathway is described in the “buffer-effect model,” according to which social support is an important resource that allows the effect of stressors to be buffered and negative consequences to be mitigated. A chronic neurodegenerative syndrome, such as dementia, can be seen as a considerable stressor, whose negative effects, including on mental health, may be buffered by a supportive social environment. Conversely, a lack of social support in this vulnerable population can add further strain, which may in turn lead to more depressive symptoms. In future studies, it would be interesting to explore the mechanisms through which social support may affect depressive symptoms in people with dementia. One approach could involve measuring the level of stress, for instance, through self-reported data or biomarkers, to investigate its potential mediating role in the relationship between social support and depressive symptoms.

Our findings contribute to the broader research on dementia and aging by addressing a critical gap in understanding the factors that are associated with depression in dementia. So far, the focus has primarily been on neurobiological mechanisms and neuropathology in Alzheimer’s disease and related dementias as the primary causes of depressive symptoms (e.g., Bennett & Thomas, 2014; Olin et al., 2002). By adopting a biopsychosocial perspective, this study highlights the significance of modifiable psychosocial factors, such as social support, in influencing mental health outcomes. This perspective opens opportunities for nonpharmacological interventions aimed at improving well-being in dementia.

An important observation in this study was that sociodemographic factors such as age, sex, and education did not predict depressive symptoms in individuals with dementia. This finding aligns with prior research (Sinclair et al., 2023; Steck et al., 2018) and suggests that the risk of depression in dementia differs from patterns observed in the general population. Instead, social support may be a particularly important predictor of depressive symptoms in this group. One possible explanation is that as dementia progresses, individuals increasingly depend on external support, making the availability and quality of social contacts more crucial for their mental health (McLaughlin et al., 2010). Additionally, the role of social support appears to shift across dementia stages. Research suggests that in early dementia, individuals value perceived independence and respect, while in later stages, companionship and direct assistance become more important (Yang et al., 2020). This highlights that social support may not only be a protective factor but also a dynamic and evolving need in dementia. Further research is needed to determine how the relationship between social support and depression differs between people with and without dementia.

Beyond social support, this study also examined cognitive and functional status as predictors of depressive symptoms. The literature on the relationship between cognitive function and depression in dementia has been inconsistent, with findings varying across studies (Steck et al., 2018). In this study, individuals with higher cognitive status exhibited more depressive symptoms, a finding that may be explained by the role of anosognosia, or the lack of awareness of cognitive deficits. Individuals in the early stages of dementia are often more conscious of their cognitive decline, which has been associated with increased depressive symptoms (Starkstein, 2014). As dementia advances and awareness of cognitive deficits diminishes, psychological distress may decrease, even as cognitive function continues to decline. Similarly, the study found that individuals with higher functional status exhibited fewer depressive symptoms, a finding consistent with previous research (Steck et al., 2018). Furthermore, functional decline over time was linked to increased depressive symptoms, underscoring the interplay between diminishing independence and worsening mental health in dementia.

These findings have significant implications for interventions and health policy. Social support could serve as a promising target for nonpharmacological interventions aimed at mitigating depressive symptoms in people with dementia. Promising approaches could include programs such as community centers, shared housing models, or neighborhood projects. At the policy level, the findings suggest a need to integrate psychosocial care into dementia treatment strategies. Current healthcare frameworks predominantly focus on the medical and cognitive aspects of dementia, often neglecting the importance of social and emotional well-being. Strengthening policies that support social interventions could help improve the well-being of individuals with dementia.

### Strengths and Limitations

Conducting extensive interviews and studies with individuals with cognitive impairment poses significant challenges, and bias in the responses cannot be ruled out. Moreover, social support and depressive symptoms, among other variables, were assessed through self-report rather than objective measures, which could potentially introduce self-report bias (Paulhus, 2017). For instance, some respondents may have provided socially desirable answers, potentially leading to an underestimation of the extent of depressive symptoms in our sample. However, in this comprehensive study, we implemented various measures to ensure a reliable and valid data collection as possible: Patients were visited by specially qualified nurses in their homes, allowing them to remain in a familiar environment, and all data collection was conducted in a structured interview format in which the nurses used a tablet to guide them through the validated questionnaires. Moreover, the study nurses conducting the home visits and interviews were specially trained in dementia care and communication for the purpose of this study. Interviews could also be paused and resumed at a later date if needed, which was done as required.

A further limitation is that the questionnaire used to measure social support was not explicitly validated for people with cognitive impairments. However, there are indications of its validity in this sample as well: in addition to validation in a representative sample, the questionnaire was also validated in a sample of patients with mental health diagnoses and in hospitalized orthopedic patients (Fydrich et al., 2007). In addition, the questionnaire has already been used in studies in people with dementia (e.g., Gellert et al., 2018). One advantage is that the questionnaire was developed and validated in Germany, making it appropriate for the cultural context.

There are also limitations regarding the generalizability of our findings. Community-dwelling individuals with dementia in the north-eastern part of Germany were recruited through their GP. It remains to be examined to what extent these results can be applied to individuals with dementia in other contexts, such as those living in nursing homes.

However, one important strength of the study is its proximity to routine care and the resulting increased external validity of the results. Another strength is that the extensive in-home interviews conducted by specially qualified nurses provide first-hand information on the psychosocial situation of people with dementia. This information is missing in many studies, which is likely why the role of the social environment for depression and neuropsychiatric symptoms in people with dementia has hardly been investigated. Another major strength of our study is that we model individual trajectories and consider the role of interindividual differences as well as intraindividual changes over time in social support for depressive symptoms. In addition, we also consider the role of between-person differences and within-person variability in cognitive and functional status for depressive symptoms in people with dementia, which, to our knowledge, has not been done before.

### Implications

Our study demonstrates that depressive symptoms in dementia are not only associated with biological factors but also with modifiable factors—specifically, the level of support from the social environment. This finding highlights the importance of prioritizing not only the medical but also the psychosocial needs of individuals with dementia. These insights could provide a valuable starting point for developing nonpharmacological intervention strategies.

## Supplementary Material

igaf047_suppl_Supplementary_Figures_1_Tables_1-2

## Data Availability

The study was not preregistered. The data presented in this study are available from the PI of the DelpHi-MV trial, WH, upon reasonable request.

## References

[CIT0001] Asmer, M. S., Kirkham, J., Newton, H., Ismail, Z., Elbayoumi, H., Leung, R. H., & Seitz, D. P. (2018). Meta-analysis of the prevalence of major depressive disorder among older adults with dementia. Journal of Clinical Psychiatry, 79, 15460. https://doi.org/10.4088/JCP.17r11772 30085437

[CIT0002] Bennett, S., & Thomas, A. J. (2014). Depression and dementia: Cause, consequence or coincidence? Maturitas, 79, 184–190. https://doi.org/10.1016/j.maturitas.2014.05.009 24931304

[CIT0033] Berkman, L. F., Glass, T., Brissette, I. & Seeman, T. E. (2000). From social integration to health: Durkheim in the new millennium. Social Science & Medicine, 51(6), 843–857. https://doi.org/10.1016/S0277-9536(00)00065-4 10972429

[CIT0003] Blotenberg, I., Boekholt, M., Michalowsky, B., Platen, M., Rodriguez, F. S., Teipel, S., Hoffmann, W., & Thyrian, J. R. (2024). What influences life expectancy in people with dementia? Social support as an emerging protective factor. Age and Ageing, 53, afae044. https://doi.org/10.1093/ageing/afae044 38497234 PMC10945357

[CIT0004] Blotenberg, I., Hoffmann, W., & Thyrian, J. R. (2023). Dementia in Germany: Epidemiology and prevention potential. Deutsches Arzteblatt international, 120, 470–476. https://doi.org/10.3238/arztebl.m2023.0100 37226316 PMC10487668

[CIT0005] Blotenberg, I., Wittström, F., Michalowsky, B., Platen, M., Wucherer, D., Teipel, S., Hoffmann, W., & Thyrian, J. R. (2025). Modifiable risk factors and symptom progression in dementia over up to 8 years–Results of the DelpHi-MV trial. Alzheimer's & Dementia (Amsterdam, Netherlands), 17(1), e70050. https://doi.org/10.1002/dad2.70050 PMC11730075 39811699

[CIT0006] Charlson, M. E., Pompei, P., Ales, K. L., & MacKenzie, C. R. (1987). A new method of classifying prognostic comorbidity in longitudinal studies: Development and validation. Journal of Chronic Diseases, 40, 373–383. https://doi.org/10.1016/0021-9681(87)90171-8 3558716

[CIT0007] Cohen, S., Underwood, L. G., & Gottlieb, B. H. (2000). Social support measurement and intervention: A guide for health and social scientists. Oxford University Press.

[CIT0008] Fydrich, T., Sommer, G., & Brähler, E. (2007). Fragebogen zur Sozialen Unterstützung (FSozU) [Social Support Questionnaire (F-SozU)]. Hogrefe.

[CIT0009] Gariépy, G., Honkaniemi, H., & Quesnel-Vallee, A. (2016). Social support and protection from depression: systematic review of current findings in Western countries. The British Journal of Psychiatry: The Journal of Mental Science, 209, 284–293. https://doi.org/10.1192/bjp.bp.115.169094 27445355

[CIT0010] Gellert, P., Häusler, A., Suhr, R., Gholami, M., Rapp, M., Kuhlmey, A., & Nordheim, J. (2018). Testing the stress-buffering hypothesis of social support in couples coping with early-stage dementia. PLoS One, 13, e0189849. https://doi.org/10.1371/journal.pone.0189849 29300741 PMC5754077

[CIT0011] Giebel, C., Talbot, C. V., Wharton, E., Lorenz-Dant, K., Suárez-González, A., Cannon, J., Tetlow, H., Lion, K. M., & Thyrian, J. R. (2023). The early impacts of COVID-19 on people living with dementia: Part I of a mixed-methods systematic review. Aging & Mental Health, 27, 533–546. https://doi.org/10.1080/13607863.2022.2084509 35763444 PMC11996062

[CIT0012] Hindmarch, I., Lehfeld, H., de Jongh, P., & Erzigkeit, H. (1998). The Bayer activities of daily living scale (B-ADL). Dementia and Geriatric Cognitive Disorders, 9, 20–26. https://doi.org/10.1159/000051195 9718231

[CIT0013] Kessler, J., Markowitsch, H. J., & Denzler, P. (1990). Mini-Mental-Status-Test (MMST) [German Version]. Beltz Test GmbH.

[CIT0014] Lyketsos, C. G., & Olin, J. (2002). Depression in Alzheimer’s disease: Overview and treatment. Biological Psychiatry, 52, 243–252. https://doi.org/10.1016/s0006-3223(02)01348-3 12182930

[CIT0015] McLaughlin, T., Feldman, H., Fillit, H., Sano, M., Schmitt, F., Aisen, P., Leibman, C., Mucha, L., Ryan, J. M., Sullivan, S. D., Spackman, D. E., Neumann, P. J., Cohen, J., & Stern, Y. (2010). Dependence as a unifying construct in defining Alzheimer’s disease severity. Alzheimer's & Dementia: The Journal of the Alzheimer's Association, 6, 482–493. https://doi.org/10.1016/j.jalz.2009.09.004 PMC3884683 21044778

[CIT0016] Michalowsky, B., Blotenberg, I., Platen, M., Teipel, S., Kilimann, I., Portacolone, E., Bohlken, J., Rädke, A., Buchholz, M., Scharf, A., Muehlichen, F., Xie, F., Thyrian, J. R., & Hoffmann, W. (2024). Clinical outcomes and cost-effectiveness of collaborative dementia care: A secondary analysis of a cluster randomized clinical trial. JAMA Network Open, 7, e2419282–e2419282. https://doi.org/10.1001/jamanetworkopen.2024.19282 38967926 PMC11227088

[CIT0017] Olin, J. T., Schneider, L. S., Katz, I. R., Meyers, B. S., Alexopoulos, G. S., Breitner, J. C., Bruce, M. L., Caine, E. D., Cummings, J. L., Devanand, D. P., Krishnan, K. R. R., Lyketsos, C. G., Lyness, J. M., Rabins, P. V., Reynolds, C. F., Rovner, B. W., Steffens, D. C., Tariot, P. N., & Lebowitz, B. D. (2002). Provisional diagnostic criteria for depression of Alzheimer disease. The American Journal of Geriatric Psychiatry: Official Journal of the American Association for Geriatric Psychiatry, 10, 125–128. https://doi.org/10.1097/00019442-200203000-00003 11925273

[CIT0018] Park, S.-H. (2022). Which of the Cornell scale for depression in dementia or the geriatric depression scale is more useful to screen for depression in older adults? Asian Journal of Psychiatry, 72, 103147. https://doi.org/10.1016/j.ajp.2022.103147 35533539

[CIT0019] Paulhus, D. L. (2017). Socially desirable responding on self-reports. In V. Zeigler-Hill & T. Shackelford (Eds.), Encyclopedia of personality and individual differences. https://doi.org/10.1007/978-3-319-28099-8_1349-1

[CIT0020] R Core Team. (2022). R: A language and environment for statistical computing. R Foundation for Statistical Computing. https://www.R-project.org/

[CIT0021] Samtani, S., Mahalingam, G., Lam, B. C. P., Lipnicki, D. M., Lima-Costa, M. F., Blay, S. L., Castro-Costa, E., Shifu, X., Guerchet, M., Preux, P. M., Gbessemehlan, A., Skoog, I., Najar, J., Rydberg Sterner, T., Scarmeas, N., Kim, K. W., Riedel-Heller, S., Röhr, S., Pabst, A., … Brodaty, H.; SHARED consortium for the Cohort Studies of Memory in an International Consortium (COSMIC). (2022). Associations between social connections and cognition: A global collaborative individual participant data meta-analysis. The Lancet Healthy Longevity, 3(11), e740–e753. https://doi.org/10.1016/S2666-7568(22)00199-4 36273484 PMC9750173

[CIT0022] Schwarzbach, M., Luppa, M., Forstmeier, S., König, H. H., & Riedel‐Heller, S. G. (2014). Social relations and depression in late life—A systematic review. International Journal of Geriatric Psychiatry, 29, 1–21. https://doi.org/10.1002/gps.3971 23720299

[CIT0023] Sinclair, L. I., Lawton, M. A., Palmer, J. C., & Ballard, C. G.; Alzheimer’s Disease Neuroimaging Initiative. (2023). Characterization of depressive symptoms in dementia and examination of possible risk factors. Journal of Alzheimer’s Disease Reports, 7, 213–225. https://doi.org/10.3233/ADR-239000 PMC10041449 36994115

[CIT0024] Starkstein, S. E. (2014). Anosognosia in Alzheimer’s disease: Diagnosis, frequency, mechanism and clinical correlates. Cortex, 61, 64–73. https://doi.org/10.1016/j.cortex.2014.07.019 25481465

[CIT0025] Steck, N., Cooper, C., & Orgeta, V. (2018). Investigation of possible risk factors for depression in Alzheimer’s disease: A systematic review of the evidence. Journal of Affective Disorders, 236, 149–156. https://doi.org/10.1016/j.jad.2018.04.034 29734098

[CIT0026] Taylor, S. E. (2011). Social support: A review. In H. S. Friedman (Ed.), The Oxford handbook of health psychology (pp. 189–214). Oxford University Press.

[CIT0027] Thyrian, J. R., Hertel, J., Wucherer, D., Eichler, T., Michalowsky, B., Dreier-Wolfgramm, A., Zwingmann, I., Kilimann, I., Teipel, S., & Hoffmann, W. (2017). Effectiveness and safety of dementia care management in primary care: A randomized clinical trial. JAMA Psychiatry, 74, 996–1004. https://doi.org/10.1001/jamapsychiatry.2017.2124 28746708 PMC5710469

[CIT0028] UNESCO. (2003). International Standard Classification of Education, ISCED 1997. Springer US.

[CIT0029] Vila, J. (2021). Social support and longevity: Meta-analysis-based evidence and psychobiological mechanisms. Frontiers in Psychology, 12, 717164. https://doi.org/10.3389/fpsyg.2021.717164 34589025 PMC8473615

[CIT0030] World Health Organization. (2021). *Global status report on the public health response to dementia*. https://iris.who.int/bitstream/handle/10665/344701/9789240033245-eng.pdf

[CIT0031] Yang, S., Zhang, Y., Xie, S., Chen, Y., Jiang, D., Luo, Y., Zhao, Q., & Yang, B. (2020). Predictors of perceived social support for patients with dementia: A mixed-methods study. Clinical Interventions in Aging, 15, 595–607. https://doi.org/10.2147/CIA.S249223 32431493 PMC7201008

[CIT0034] Yesavage, J. A., Brink, T. L., Rose, T. L., Lum, O., Huang, V., Adey, M., & Leirer, V. O. (1982). Development and validation of a geriatric depression screening scale: A preliminary report. Journal of Psychiatric Research, 17(1), 37–49. https://doi.org/10.1016/0022-3956(82)90033-4 7183759

