# Supplementary material for: The Role of Social Support for Depressive Symptoms in Dementia: A Four-Year Longitudinal Study
Source: Innov Aging. 2025 Jul 1;9(6):igaf047. doi: 10.1093/geroni/igaf047 (PMC12210947; doi:10.1093/geroni/igaf047)
Supplement: igaf047_suppl_Supplementary_Figures_1_Tables_1-2 [file igaf047_suppl_supplementary_figures_1_tables_1-2.docx]

*Innovation in Aging* Supplemental Material: Blotenberg et al. The Role of Social Support for Depressive Symptoms in Dementia: A Four-Year Longitudinal Study.

Supplementary Figure 1. Participant flow over four years.


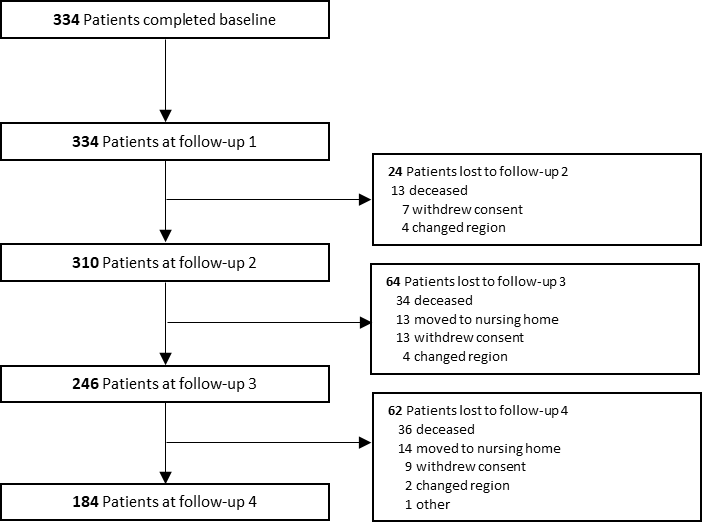


**Supplementary Table 1.** Prediction of depressive symptoms across four years (unimputed data).

| **Variable** | | **Estimate (log)** | | |  | **% change** | | | ***p*** | |
| --- | --- | --- | --- | --- | --- | --- | --- | --- | --- | --- |
|  |  |  | **95% CI (lower)** | **95% CI  (upper)** |  |  | **95% CI (lower)** | **95% CI  (upper)** |  | |
| *Model 1. Null model* | |  |  |  |  |  |  |  |  |  |
| Fixed effects | |  |  |  |  |  |  |  |  |  |
|  | **Intercept** | 1.241 | 1.175 | 1.308 |  | **-** | **-** | **-** | **<0.001** | *** |
|  | **Time** | **0.03** | **0.009** | **0.051** |  | **3.045** | **0.904** | **5.232** | **0.006** | ** |
| Random effects | |  |  |  |  |  |  |  |  |  |
|  | Intercept (person) | 0.23 |  |  |  |  |  |  |  |  |
|  | Time | 0.01 |  |  |  |  |  |  |  |  |
|  | Intercept (person) x Time | -0.36 |  |  |  |  |  |  |  |  |
|  | Intercept (GP) | 0.01 |  |  |  |  |  |  |  |  |
|  | Residual | 0.15 |  |  |  |  |  |  |  |  |
|  | ICC | 0.62 |  |  |  |  |  |  |  |  |
|  | Marginal R²/ Conditional R² | 0.004 / 0.617 |  |  |  |  |  |  |  |  |
| *Model 2. Full model* | |  | | |  |  | | |  |  |
| Fixed effects | |  |  |  |  |  |  |  |  |  |
|  | **Intercept** | **2.668** | **1.478** | **3.858** |  | **-** | **-** | **-** | **<0.001** | *** |
|  | Time | -0.042 | -0.551 | 0.467 |  | -4.113 | -42.363 | 59.520 | 0.872 |  |
|  | Age | -0.001 | -0.013 | 0.012 |  | -0.100 | -1.292 | 1.207 | 0.907 |  |
|  | Sex (ref: male) | 0.040 | -0.089 | 0.17 |  | 4.081 | -8.515 | 18.530 | 0.541 |  |
|  | Living alone (ref: no) | -0.017 | -0.146 | 0.112 |  | -1.686 | -13.584 | 11.851 | 0.795 |  |
|  | Moderate education (ref: low) | -0.025 | -0.189 | 0.138 |  | -2.469 | -17.221 | 14.798 | 0.760 |  |
|  | High education (ref: low) | 0.064 | -0.308 | 0.437 |  | 6.609 | -26.508 | 54.806 | 0.734 |  |
|  | Group allocation (ref: CAU) | -0.015 | -0.167 | 0.136 |  | -1.489 | -15.380 | 14.568 | 0.841 |  |
|  | Caregiving network (ref: none) | -0.096 | -0.476 | 0.285 |  | -9.154 | -37.874 | 32.976 | 0.623 |  |
|  | Charlson comorbidity index | 0.024 | -0.002 | 0.051 |  | 2.429 | -0.200 | 5.232 | 0.072 |  |
|  | **Cognitive status (between-person differences)** | **0.033** | **0.015** | **0.051** |  | **3.355** | **1.511** | **5.232** | **<0.001** | *** |
|  | Cognitive status (within-person variability) | -0.008 | -0.027 | 0.011 |  | -0.797 | -2.664 | 1.106 | 0.408 |  |
|  | **Functional status (between-person differences)** | **-0.106** | **-0.144** | **-0.069** |  | **-10.058** | **-13.411** | **-6.667** | **<0.001** | *** |
|  | **Functional status (within-person variability)** | **-0.050** | **-0.082** | **-0.018** |  | **-4.877** | **-7.873** | **-1.784** | **0.002** | ** |
|  | **Social support (between-person differences)** | **-0.014** | **-0.02** | **-0.009** |  | **-1.390** | **-1.980** | **-0.896** | **<0.001** | *** |
|  | **Social support (within-person variability)** | **-0.009** | **-0.016** | **-0.002** |  | **-0.896** | **-1.587** | **-0.200** | **0.009** | ** |
|  | Time x age | 0.000 | -0.005 | 0.006 |  | 0.000 | -0.499 | 0.602 | 0.914 |  |
|  | Time x sex | -0.003 | -0.058 | 0.052 |  | -0.300 | -5.635 | 5.338 | 0.921 |  |
|  | Time x living alone | -0.012 | -0.065 | 0.042 |  | -1.193 | -6.293 | 4.289 | 0.671 |  |
|  | Time x moderate education | 0.019 | -0.049 | 0.087 |  | 1.918 | -4.782 | 9.090 | 0.583 |  |
|  | ***Time x high education*** | ***-0.154*** | ***-0.326*** | ***0.018*** |  | ***-14.273*** | ***-27.819*** | ***1.816*** | ***0.079*** |  |
|  | Time x group allocation (ref: CAU) | -0.007 | -0.061 | 0.046 |  | -0.698 | -5.918 | 4.707 | 0.790 |  |
|  | Time x caregiving network | 0.025 | -0.131 | 0.18 |  | 2.532 | -12.278 | 19.722 | 0.755 |  |
|  | ***Time x Charlson comorbidity index*** | ***0.011*** | ***-0.001*** | ***0.022*** |  | ***1.106*** | ***-0.100*** | ***2.224*** | ***0.070*** |  |
|  | ***Time x cognitive status (between-person differences)*** | -0.002 | -0.01 | 0.006 |  | -0.200 | -0.995 | 0.602 | 0.591 |  |
|  | Time x cognitive status (within-person variability) | 0.002 | -0.008 | 0.012 |  | 0.200 | -0.797 | 1.207 | 0.712 |  |
|  | **Time x functional status (between-person differences)** | **0.017** | **0.001** | **0.033** |  | **1.715** | **0.100** | **3.355** | **0.043** | * |
|  | Time x functional status (within-person variability) | -0.003 | -0.02 | 0.015 |  | -0.300 | -1.980 | 1.511 | 0.755 |  |
|  | Time x social support (between-person differences) | -0.002 | -0.004 | 0.001 |  | -0.200 | -0.399 | 0.100 | 0.143 |  |
|  | Time x social support (within-person variability) | 0.001 | -0.003 | 0.004 |  | 0.100 | -0.300 | 0.401 | 0.770 |  |
| Random effects | |  |  |  |  |  |  |  |  |  |
|  | Intercept (person) | 0.14 |  |  |  |  |  |  |  |  |
|  | Time | 0.01 |  |  |  |  |  |  |  |  |
|  | Intercept x Time | -0.22 |  |  |  |  |  |  |  |  |
|  | Intercept (GP) | 0.01 |  |  |  |  |  |  |  |  |
|  | Residual | 0.15 |  |  |  |  |  |  |  |  |
|  | ICC | 0.51 |  |  |  |  |  |  |  |  |
|  | Marginal R²/ Conditional R² | 0.222 / 0.619 |  |  |  |  |  |  |  |  |

*Notes*. GP = General Practitioner, CI = Confidence Interval, ICC = Intraclass Correlation Coefficient

**Supplementary Table 2.** Prediction of depression across four years (imputed data).

| **Variable** | | **OR** | | | ***p*** |  |
| --- | --- | --- | --- | --- | --- | --- |
|  |  |  | **95% CI**  **(lower)** | **95% CI  (upper)** |  |  |
| *Model 3. Full model* | |  |  |  |  |  |
| Fixed effects | |  |  |  |  |  |
|  | **Intercept** | **-** | **-** | **-** | **<0.001** | *** |
|  | **Time** | 0.179 | 0.019 | 1.660 | 0.130 |  |
|  | Age | 0.935 | 0.861 | 1.016 | 0.113 |  |
|  | Sex (ref: male) | 1.135 | 0.455 | 2.832 | 0.787 |  |
|  | Living alone (ref: no) | 0.797 | 0.319 | 1.993 | 0.628 |  |
|  | Moderate education (ref: low) | 0.870 | 0.294 | 2.579 | 0.802 |  |
|  | High education (ref: low) | 0.242 | 0.015 | 3.857 | 0.315 |  |
|  | Group allocation (ref: CAU) | 1.217 | 0.463 | 3.199 | 0.690 |  |
|  | Caregiving network (ref: none) | 0.381 | 0.043 | 3.342 | 0.384 |  |
|  | Charlson comorbidity index | 1.037 | 0.871 | 1.235 | 0.685 |  |
|  | **Cognitive status (between-person differences)** | **1.180** | **1.056** | **1.318** | **0.003** | ** |
|  | Cognitive status (within-person variability) | 1.070 | 0.969 | 1.181 | 0.184 |  |
|  | **Functional status (between-person differences)** | **0.612** | **0.471** | **0.795** | **<0.001** | *** |
|  | **Functional status (within-person variability)** | **0.785** | **0.644** | **0.956** | **0.016** | * |
|  | **Social support (between-person differences)** | **0.943** | **0.903** | **0.985** | **0.008** | ** |
|  | **Social support (within-person variability)** | **0.961** | **0.925** | **0.998** | **0.039** | *** |
|  | **Time x age** | **1.031** | **1.007** | **1.057** | **0.012** | ** |
|  | **Time x sex** | 0.834 | 0.639 | 1.090 | 0.184 |  |
|  | Time x living alone | 1.008 | 0.772 | 1.318 | 0.951 |  |
|  | Time x moderate education | 0.934 | 0.680 | 1.283 | 0.673 |  |
|  | ***Time x high education*** | 1.193 | 0.531 | 2.683 | 0.669 |  |
|  | Time x group allocation (ref: CAU) | 0.925 | 0.698 | 1.227 | 0.590 |  |
|  | Time x caregiving network | 1.142 | 0.599 | 2.176 | 0.686 |  |
|  | Time x Charlson comorbidity index | 1.020 | 0.969 | 1.073 | 0.449 |  |
|  | **Time x cognitive status (between-person differences)** | ***0.971*** | ***0.940*** | ***1.003*** | ***0.078*** |  |
|  | Time x cognitive status (within-person variability) | 0.976 | 0.950 | 1.003 | 0.084 |  |
|  | **Time x functional status (between-person differences)** | **1.110** | **1.028** | **1.199** | **0.008** | * |
|  | ***Time x functional status (within-person variability)*** | ***1.054*** | ***0.992*** | ***1.120*** | ***0.091*** |  |
|  | Time x social support (between-person differences) | 0.996 | 0.983 | 1.009 | 0.555 |  |
|  | Time x social support (within-person variability) | 0.994 | 0.982 | 1.007 | 0.396 |  |
| Random effects | |  |  |  |  |  |
|  | Intercept (person) | 5.08 |  |  |  |  |
|  | Time | 0.18 |  |  |  |  |
|  | Intercept x Time | -1.00 |  |  |  |  |
|  | Intercept (GP) | 0.00 |  |  |  |  |
|  | Residual | 3.29 |  |  |  |  |
|  | ICC | 0.41 |  |  |  |  |
|  | Marginal R²/ Conditional R² | 0.279 / 0.577 |  |  |  |  |

*Notes.* GP = General Practitioner, CI = Confidence Interval, ICC = Intraclass Correlation Coefficient. Depression defined as Geriatric Depression Scale (GDS) Score > 5.
